# Real-world experience of intravenous ferric derisomaltose evaluated through safety and efficacy reporting in the UK

**DOI:** 10.1038/s41598-022-23581-3

**Published:** 2022-11-07

**Authors:** Rhona C. F. Sinclair, Sean Nadaraja, Nicholas A. Kennedy, Mai Wakatsuki, Sunil Bhandari

**Affiliations:** 1 Department of Anaesthesia, Royal Victoria Infirmary, The Newcastle Upon Tyne Hospitals NHS Foundations Trust, Newcastle Upon Tyne, UK; 2 Royal National Orthopaedic Hospital NHS Trust, London, UK; 3 Department of Gastroenterology, Royal Devon and Exeter NHS Foundation Trust, Exeter, UK; 4 Shackleton Department of Anaesthesia, University Hospital Southampton NHS Foundation Trust, Southampton, UK; 5 Department of Renal Medicine, Hull University Teaching Hospitals NHS Trust, Kingston Upon Hull, HU3 2JZ UK

**Keywords:** Health care, Medical research

## Abstract

Ferric derisomaltose (FDI; Monofer) is used in clinical practice to treat iron deficiency, but the safety and efficacy of FDI has not been robustly evaluated in a large real-world study. This retrospective, multicentre, audit-based, observational study provides pragmatic information about safety and clinical responses with FDI across therapy areas and patient populations, helping to facilitate treatment decisions. Participating sites provided data from the medical records of adults who had received ≥ 1 FDI infusion. The primary outcome was the incidence of adverse reactions within 24 hours of the FDI infusion. Secondary outcomes included the change from baseline in haemoglobin and ferritin up to 12 months post infusion. In total, 19 sites provided data for a total of 7354 FDI-treated patients; 64.3% of patients were female, and 42.2% were aged ≥ 70 years. Surgery was the main hospital specialty (34.5%). The incidence of any recorded adverse reactions, hypersensitivity reactions, and anaphylaxis were 1.7%, 0.4%, and < 0.1%, respectively, regardless of baseline anaemia status. Statistically significant increases in haemoglobin and ferritin were observed between baseline and Month 4 following FDI treatment (p < 0.0001). Improvements in haemoglobin were more pronounced for hospital specialties where operative blood loss is expected (surgery/obstetrics) compared with those where blood loss is not expected. This study provides real-world clinical evidence for the low risk of adverse reactions with FDI across diverse patient populations, providing reassurance that intravenous iron is not associated with serious toxicity. These findings may inform changes in intravenous iron delivery to provide effective therapy to more patients in need.

## Introduction

Clinical guidelines recommend the use of intravenous iron to treat iron deficiency with or without anaemia across different hospital specialty areas, including cardiology^1^, patient blood management^2,3^, nephrology^4–6^, gastroenterology^7,8^, and obstetrics^9^.

Ferric derisomaltose (FDI) is an intravenous iron formulation approved in Europe (Monofer/Monoferric/Monover/Monofar), the US and Canada (Monoferric), and China (Monofer) for iron repletion in a single visit where oral iron is not effective or is not tolerated.

The efficacy and safety of FDI have been confirmed in randomised controlled trials (RCTs) across different therapy areas^10–19^. Other investigations of FDI conducted in real-world clinical settings (i.e., observational studies, service evaluations) also report good efficacy and safety profiles, including significantly better responses (haemoglobin and iron parameters) where dosing was optimised based on the iron need of the individual^20–22^. However, none of these investigations included an adequate number of patients to allow for robust characterisation of the efficacy and safety profile of FDI in the real-world setting, nor did they span across different therapy areas. Many centres in the UK collect local data to inform and regulate clinical practice, but much of these data are unpublished. Consequently, there is an opportunity to pool the data that are routinely collected by UK centres on the use of intravenous iron in clinical care, and use it to inform the medical community on the safety and efficacy of FDI in the real-world setting.

The principal aim of this retrospective, multicentre, audit-based, observational study was to report safety and efficacy data for FDI in diverse clinical patient groups in the UK. The study provides pragmatic information about safety and clinical responses to FDI across therapy areas and patient populations, helping to facilitate future treatment decisions.

## Methods

### Data collection

Potential sites were identified based on: 1) FDI sales of more than 150 g (approximately 100 patient doses) in the previous 12 months (April 2019 to March 2020), selected to ensure inclusion of a sufficient number of sites; and/or 2) a record of publishing data on FDI (abstracts/manuscripts). These sites were invited, by email, to participate in the study and contribute data. Each site was contacted a minimum of three times, by email, to encourage participation. Eligible data were obtained from the medical records of adult patients (aged ≥ 18 years) who had received ≥ 1 infusion of FDI. Previously published data (including data published in abstract form only), and unpublished data held on local databases, were also considered eligible.

The minimum requirement for data from sites was haemoglobin level prior to FDI treatment and the dose of FDI administered. A full list of the data parameters that could be submitted are presented in Supplemental Table S4.

Local audit data were anonymised, allocated a local identification number based on the hospital and/or the clinician (for sites with more than one intravenous iron service), and uploaded to a secure workspace (Box secure file sharing [International Organization for Standardization 27001 certified]; CA, USA) — all tasks were managed by a third party (Plus-Project Ltd). Within this workspace, each site was designated a folder for the transfer of data that could be accessed only by Plus-Project staff and the clinical lead at each site. All data handling was performed according to global data protection and UK National Health Service (NHS) guidelines.

Data were collected retrospectively, outside of a controlled clinical research setting and, therefore, it is acknowledged that parts of the dataset may be inconsistent and/or incomplete.

### Outcomes

The primary outcome was the incidence of recorded adverse reactions within 24 hours of the FDI infusion, defined as the composite of anaphylaxis, hypersensitivity reactions (HSRs), extravasation, Fishbane reactions, and other reactions deemed to be related to FDI administration. Extravasation (i.e., paravenous leakage) can lead to irritation and long-lasting staining of the skin^23^. Fishbane reactions are characterised by flushing in the face, acute chest and/or back pain, and chest tightness sometimes with dyspnoea^23^. Other reactions encompassed any isolated symptoms that were not consistent with a specific reaction. Due to the challenges of identifying anaphylaxis in a consistent manner between centres, study sites reporting cases of possible anaphylaxis were sent a follow-up questionnaire to capture further information relating to the symptoms experienced by the patient and the intervention required (Supplemental Table S5), such that these events could be validated and accurately categorised. The information received was reviewed by two members of the investigational team and, where necessary, the anaphylaxis event was recategorised into one of the other four groups described above. In addition to the primary outcome, the reaction rate for anaphylaxis was calculated (number of reactions/total number of infusions × 1000), as was the proportion of patients experiencing adverse reactions, by year.

The secondary outcomes included the change in haemoglobin and ferritin from baseline to Days 7, 14, and 28, and to 4, 6, and 12 months after FDI infusion.

### Statistics

All available data from eligible patients receiving FDI were included in the analyses; there was no imputation of missing data. Demographics and baseline characteristics were summarised descriptively. Summary statistics for change in haemoglobin and ferritin over time were produced. The absolute change in haemoglobin and ferritin from baseline to Month 4 was analysed using an analysis of covariance (ANCOVA) model, adjusted for dose and baseline value. The 4-month timepoint is of most relevance to clinical practice (as this is a routine follow-up timepoint and when additional iron may be required in many patient populations, e.g., those with chronic kidney disease) and provided most of the available data. Certain data parameters were analysed according to various pre-specified subgroups — gender, age category, baseline haemoglobin, baseline ferritin, anaemia status, and hospital specialty. The potential influence of blood loss on the change in haemoglobin was explored through further analysis of the data according to the hospital specialties where a discrete episode of blood loss can be expected (i.e., surgery and obstetrics) and where this type of blood loss is not expected (all other specialties). The safety analyses included data for all treatment courses of FDI, whereas the efficacy analyses were conducted, primarily, for the first treatment course only. A treatment course was defined as the delivery of the prescribed total iron dose in one administration, or as two infusions separated by at least 1 week. Statistical analyses were performed using SAS Version 9.4.

### Study approval

The protocol was considered by the Research and Development Team at Hull University Teaching Hospitals, and by the Caldicott Guardian, and was approved as an extended audit (reference number: 2020.082). The Caldicott Guardian confirmed that further Regional Ethics Committee (REC) approval was not required. Where required by the local Trust, the hospital transferring data to the Box workspace had to obtain approval to share data under the Caldicott agreement. All methods were performed in accordance with the relevant guidelines and regulations. Consent to participate and consent for publication were not required as this was a retrospective study of patient records.

## Results

### Patient population

**Figure 1:**
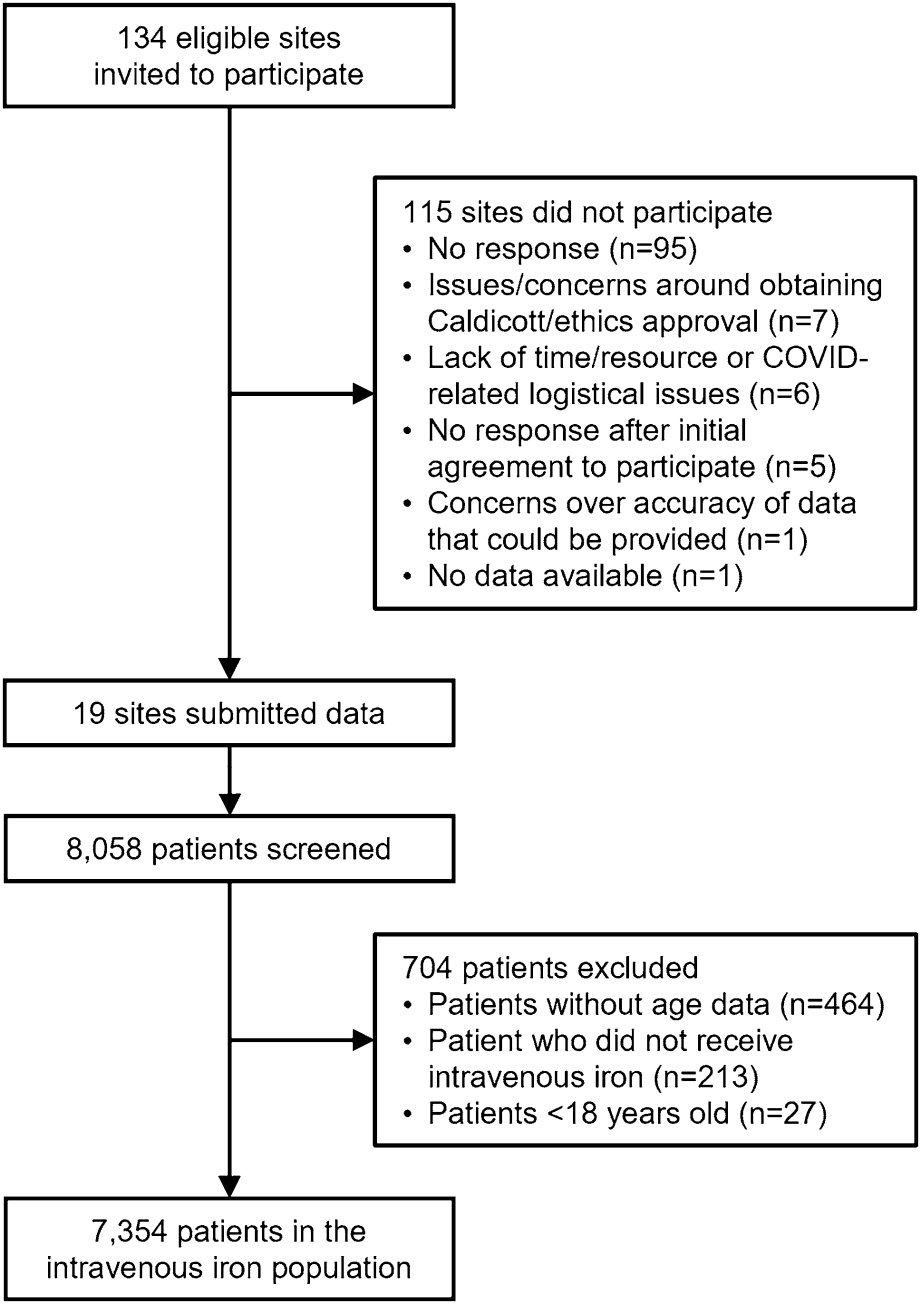
Patient disposition. *COVID* coronavirus disease.

Figure 1 presents the overall patient disposition; a breakdown by participating centre is presented in Supplemental Table S1.

Baseline characteristics for the overall population receiving intravenous iron are presented in Table 1 (and by baseline anaemia status in Supplemental Table S2). There were more female than male patients (64.3% and 35.7%, respectively) and there was a high proportion of patients ≥ 70 years (42.2%). There was a higher proportion of females than males in the 18–49 years and 50–69 years age groups; the proportions were more balanced in the older age groups (≥ 70 years) (Supplemental Fig. S1). Surgery was the main hospital specialty (34.5%) followed by nephrology (18.9%) and gastroenterology (13.5%).

**Table 1:** Baseline characteristics.

	All intravenous iron patients (N = 7354)	Female (N = 4500)	Male (N = 2502)
**Age, years**	(n = 7118)	(n = 4326)	(n = 2440)
Mean (SD)	60.6 (20.5)	57.4 (20.6)	69.8 (15.6)
**Age group, n (%)**	(n = 7118)	(n = 4326)	(n = 2440)
18–49 years	2343 (31.9)	1742 (38.7)	301 (12.0)
50–69 years	1669 (22.7)	1015 (22.6)	642 (25.7)
70–79 years	1556 (21.2)	771 (17.1)	767 (30.7)
≥ 80 years	1550 (21.1)	798 (17.7)	730 (29.2)
**Weight, kg**	(n = 3160)	(n = 1560)	(n = 1263)
Mean (SD)	76.9 (19.6)	72.8 (19.3)	83.5 (18.6)
**Hospital specialty, n (%)**			
Cardiology	93 (1.3)	40 (0.9)	53 (2.1)
Gastroenterology	990 (13.5)	611 (13.6)	376 (15.0)
Gynaecology	174 (2.4)	174 (3.9)	0 (0.0)
Haematology	480 (6.5)	369 (8.2)	111 (4.4)
Nephrology	1393 (18.9)	635 (14.1)	757 (30.3)
Obstetrics	814 (11.1)	517 (11.5)	0 (0.0)
Oncology	26 (0.4)	11 (0.2)	15 (0.6)
Other medicine^a^	850 (11.6)	683 (15.2)	167 (6.7)
Surgery^b^	2534 (34.5)	1460 (32.4)	1023 (40.9)
**Anaemia status, n (%)**^**c**^	(n = 6899)	(n = 4289)	(n = 2313)
Anaemic patients	6217 (84.5)	3730 (82.9)	2217 (88.6)
Non-anaemic patients	682 (9.3)	559 (12.4)	96 (3.8)
**Haemoglobin (g/L)**	(n = 6954)	(n = 4289)	(n = 2313)
Mean (SD)	99.6 (17.6)	98.6 (17.9)	102.4 (17.3)
**Haemoglobin level, n (%)**	(n = 6954)	(n = 4289)	(n = 2313)
> 130 g/L	192 (2.6)	105 (2.3)	86 (3.4)
> 120 to ≤ 130 g/L	616 (8.4)	374 (8.3)	241 (9.6)
> 110 to ≤ 120 g/L	1144 (15.6)	702 (15.6)	431 (17.2)
> 100 to ≤ 110 g/L	1432 (19.5)	825 (18.3)	519 (20.7)
> 90 to ≤ 100 g/L	1439 (19.6)	848 (18.8)	457 (18.3)
> 80 to ≤ 90 g/L	1118 (15.2)	716 (15.9)	331 (13.2)
≤ 80 g/L	1013 (13.8)	719 (16.0)	248 (9.9)
**Ferritin (μg/L)**	(n = 5916)	(n = 3665)	(n = 1970)
Mean (SD)	62.8 (149.6)	50.3 (123.8)	92.3 (192.5)
**Ferritin level, n (%)**	(n = 5916)	(n = 3665)	(n = 1970)
< 30 µg/L	3785 (51.5)	2560 (56.9)	967 (38.6)
≥ 30 to ≤ 100 µg/L	1190 (16.2)	667 (14.8)	509 (20.3)
> 100 µg/L	941 (12.8)	438 (9.7)	494 (19.7)

The proportions presented in the table were calculated using the overall number of patients as the denominator for each group (N = 7354 for the ‘All intravenous iron patients’ group; N = 4500 for the female group; N = 2502 for the male group).

‘Race’ have not been reported as the data were missing for most patients (88.8% [n = 6529]).

*N* number of patients, *n* number of patients with data, *SD* standard deviation.

^a^‘Other medicine’ included the following terms: ‘Emergency Department’, ‘Geriatric’, ‘General Practice’, ‘General Medicine’, ‘Hepatology’, ‘Medicine’, ‘Other’, ‘Rheumatology’, ‘Unclear’; it also captures patients for whom no specialty was provided.

^b^‘Surgery’ included the following terms: ‘Anaesthesia/Anaesthetics’, ‘Breast’, ‘Colorectal’, ‘Ear, nose, and throat’, ‘Orthopaedics’, ‘Pre-operative’, ‘Surgery/Surgical’, ‘Urology’, ‘Vascular’.

^c^Anaemia at baseline was defined as a haemoglobin < 130 g/L for men, < 120 g/L for women, and < 105 g/L for obstetrics patients.

### Iron dosing and treatment routine

Of the 7354 patients receiving intravenous iron, 7350 (99.9%) patients received a first course of known dose of FDI treatment (the actual dose administered during the first course was not recorded for four patients), and 425 (5.8%) patients went on to receive a second course or more. The mean (standard deviation [SD]) total iron dose administered during the first course was 1272.0 (348.12) mg, and 98.7% of patients received their first course in one infusion. Most patients (61.4%) received > 1000 mg FDI during the first course, and 38.6% of patients received ≤ 1000 mg FDI. During the first course of treatment, patients who were anaemic at baseline received a statistically significantly higher mean total dose of FDI than those who were non-anaemic (1283.0 [95% confidence interval {CI} 1274.4, 1291.7] mg versus 1198.2 [95% CI 1170.1, 1226.3] mg, respectively; p < 0.0001). Similarly, patients with a baseline haemoglobin level < 100 g/L received a statistically significantly higher mean total dose of FDI than those with a haemoglobin level ≥ 100 g/L (1308.9 [95% CI 1297.2, 1320.5] mg versus 1241.7 [95% CI 1230.2, 1253.3] mg, respectively; p < 0.0001). A summary of FDI dosing according to baseline haemoglobin level for the first treatment course is presented in Supplemental Table S3.

### Safety

A summary of the adverse reactions reported following FDI treatment (all courses) is presented in Table 2. Adverse reactions were reported for 2.2% of patients who were non-anaemic at baseline and for 1.7% of those who were anaemic at baseline. The overall rate of HSRs was low (0.4%). The incidence of HSRs was numerically higher for non-anaemic patients than for anaemic patients (0.9% versus 0.3%). Where the severity of the HSR was reported, the event was predominantly mild or moderate in nature. The proportion of patients with a clinician-validated report of anaphylaxis was low (≤ 0.1%), making it difficult to compare between the groups of patients who were anaemic and non-anaemic at baseline (Table 2). The overall reaction rate for anaphylaxis (number of reactions/total number of infusions) was 0.6 per 1000 infusions. All cases of anaphylaxis resolved, and no treatment-related deaths were reported. The proportion of patients experiencing adverse reactions over time is presented in Supplemental Fig. S2.

**Table 2:** Adverse reactions following FDI treatment (all courses).

	All intravenous iron patients (N = 7354)	Anaemic patients (N = 6217)^a^	Non-anaemic patients (N = 682)^a^
**Any adverse reaction, n (%)**	124 (1.7)	103 (1.7)	15 (2.2)
**HSRs, n (%)**	27 (0.4)	20 (0.3)	6 (0.9)
**Anaphylaxis, n (%)**	8 (0.1)	5 (< 0.1)	2 (0.3)
Clinician-validated events^b^	5 (< 0.1)	3 (< 0.1)	1 (0.1)
**Extravasation, n (%)**	5 (< 0.1)	5 (< 0.1)	0 (0.0)
**Fishbane reactions, n (%)**	54 (0.7)	50 (0.8)	2 (0.3)
**Other reaction deemed related to drug administration, n (%)**	37 (0.5)	26 (0.4)	8 (1.2)

*HSR* hypersensitivity reaction, *N* number of patients, *n* number of patients with event.

^a^Anaemia status at baseline was unknown for 455 patients.

^b^The data presented reflect the classification of events after the process of review and validation by clinicians, during which some anaphylaxis events were recategorised into one of the other four groups.

### Efficacy

**Figure 2:**
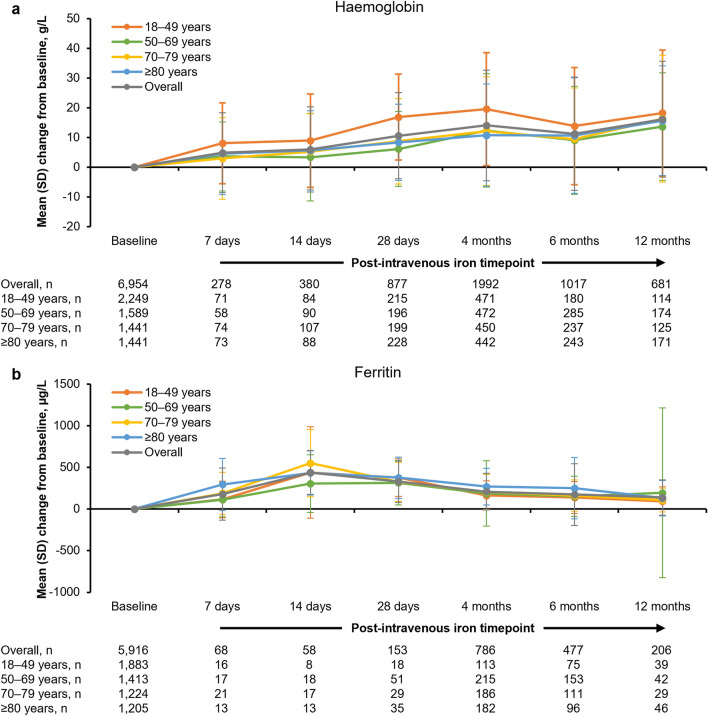
Change from baseline in haemoglobin and ferritin following first course of FDI, by patient age. Baseline mean (SD) data for all intravenous iron patients: haemoglobin = 99.6 (17.56) g/L; ferritin = 62.8 (149.64) μg/L. For all age groups, the increase in haemoglobin and ferritin versus baseline was statistically significant at 4 months (p < 0.0001). *SD* standard deviation.

Following the first course of FDI treatment, the haemoglobin level increased by a mean (SD) value of 14.1 (18.65) g/L from baseline to Month 4; the improvement in haemoglobin was most pronounced in the 18–49 years age group (Fig. 2a). For all age groups, the increase in haemoglobin versus baseline was statistically significant at 4 months (p < 0.0001). Overall, the mean (SD) increase from baseline in ferritin level was 209.6 (271.64) μg/L at Month 4 (Fig. 2b); absolute levels peaked 14 days after FDI treatment in all age groups, with statistically significantly higher levels than baseline at 4 months (p < 0.0001).

**Figure 3:**
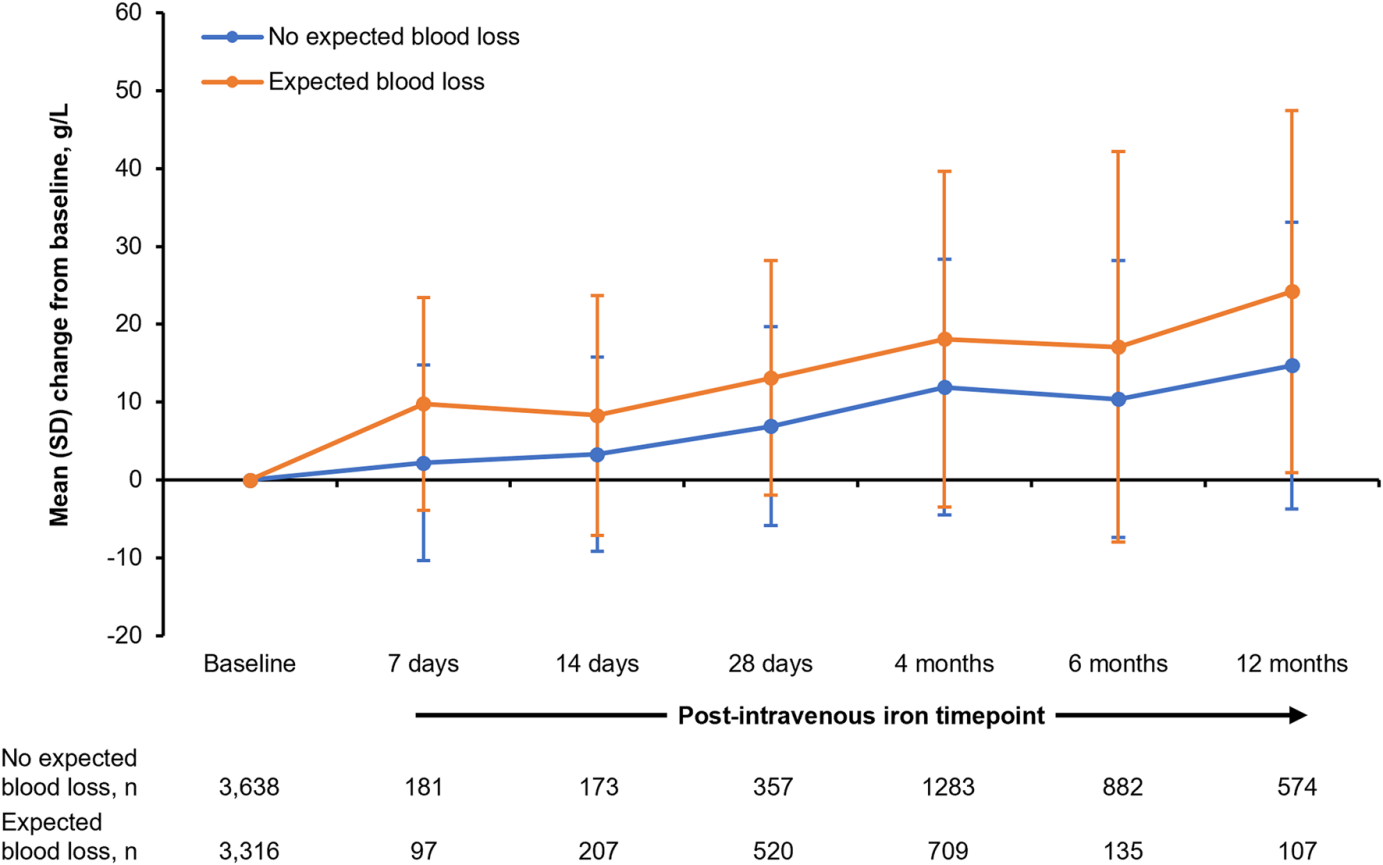
Change in haemoglobin following first course of FDI, according to the expectation of blood loss. Baseline mean (SD) data for ‘Expected blood loss’: haemoglobin = 101.3 (15.97) g/L; baseline mean (SD) data for ‘No expected blood loss’: haemoglobin = 98.0 (18.76) g/L. ‘Expected blood loss’ includes the specialties of surgery and obstetrics; ‘No expected blood loss’ includes all other hospital specialties. In both groups, the increase in haemoglobin between baseline and Month 4 was statistically significant (p < 0.0001). *SD* standard deviation.

Further analysis to examine the potential influence of blood loss on the change in haemoglobin showed that increases in haemoglobin were greater where blood loss was expected (Fig. 3). Between baseline and Month 4, there was a mean (SD) increase in haemoglobin of 18.1 (21.57) g/L in the expected blood loss group (p < 0.0001), and of 11.9 (16.42) g/L in the no expected blood loss group (p < 0.0001).

## Discussion

This study reports on the real-world experience of using FDI across diverse patient populations in the UK. The observed incidence of adverse reactions for all patients receiving intravenous iron is low: 1.7% for all adverse reactions, 0.4% for HSRs, and < 0.1% for adjudicated anaphylaxis. The results presented are consistent with previously published evaluations of FDI across various therapeutic areas^20–22,24,25^. In addition, the low incidence of HSRs reported (0.4%) provides real-world evidence to support the data generated from RCTs that were designed to evaluate the incidence of HSRs as primary or secondary endpoints^17–19,26^. The findings of this study indicate that the current generation of intravenous iron products, such as FDI, are safe for use in the real-world clinical setting, in contrast to the historical concerns with respect to first-generation products^27^.

Both anaemic and non-anaemic patients were included in this study. Anaemic patients received a statistically significantly higher mean total dose of FDI than non-anaemic patients (as did patients with a baseline haemoglobin < 100 g/L versus ≥ 100 g/L); however, the differences in dose between the two groups were not considered to be clinically important. This lack of a clinically relevant difference in FDI dosing may be due to the variation in the dosing regimens used between and within different study centres (additional information on dosing was captured by an online survey). Indeed, a variety of different dosing regimens were used by the participating hospitals — Ganzoni formula, simplified dosing, and/or fixed dosing^23^ — leading to variations in dosing throughout the dataset. Although the incidence of adverse reactions between anaemic and non-anaemic patients was numerically similar (1.7% and 2.2%, respectively), there was a lower incidence of HSRs in the anaemic group (0.3%) versus the non-anaemic group (0.9%).

There are important clinical implications of the overall low incidence of adverse reactions, including anaphylaxis and HSRs observed in this study. Historically, clinicians have been reluctant to use intravenous iron products on a regular basis, partially fuelled by the high rates of anaphylaxis and adverse events that have been reported with first-generation iron products^28^. The present study reports a low incidence of adverse reactions with FDI. This enables the reaction rate to be compared with other commonly prescribed medications, providing context for the patient ‘risk’ associated with FDI. For example, the frequency of HSRs in patients treated with intravenous penicillin is reported to be ‘high’ at 1 to 10%^29^. For other intravenous medications, the frequency of HSRs has been reported as > 10% (‘very high’) for selected monoclonal antibodies, 0.1 to < 1% (‘moderate’) for selected antibiotics, and 0.01 to < 0.1% (‘low’) for selected analgesics/non-steroidal anti-inflammatory drugs^29^. When compared to these medications, the rates of adverse reactions (1.7%) and anaphylaxis (< 0.1%) with FDI are reassuring for patients and clinicians.

The efficacy of FDI used in a non-trial setting was confirmed by the data presented from the clinical audit datasets of various hospitals used in this analysis. High-dose FDI was associated with a median increase in haemoglobin of 14.1 g/L when evaluated 4 months after treatment. Interestingly, the improvement in haemoglobin was greater for the surgery/obstetrics group (where a discrete episode of blood loss can be expected) than was observed for the other hospital specialties combined (where no blood loss is expected). In some cases, for example, in patients with inflammatory bowel disease, it may be that the surgical procedure prevented further blood loss, allowing for a greater net increase in haemoglobin following intravenous iron therapy. However, due to the lack of granularity in the data collected (i.e., types of surgery performed, use of blood transfusions, whether there were any events of obstetric-related blood loss, etc.), it is not possible to unravel further potential explanations for the observed difference.

The limitations of the study include evaluation of a single intravenous iron product, heterogeneous patient populations, different methods for selecting patients, and a variety of different dosing regimens used to prescribe FDI. The study was designed pragmatically to gather the available unpublished clinical information and, as such, it was accepted (during study design) that there was no control/comparison group, the data were not standardised, sensitivity analyses were not conducted, and that there would be ‘missing data’ (no missing data imputations were applied). Despite these limitations, we present valuable data representing real-world intravenous iron use in diverse clinical practices across the UK.

## Conclusions

In conclusion, the safety and efficacy of high-dose FDI treatment in routine clinical practice have been confirmed. This real-world study provides reassurance of the safety of intravenous iron use in the treatment of iron deficiency across a broad spectrum of patients.

## Supplementary Information

Supplementary Information.

## Data Availability

The data that support the findings of this study may be available from the local investigators upon reasonable request (via the corresponding author), if permitted according to their local governance procedures.
