# Supplementary material for: Real-world experience of intravenous ferric derisomaltose evaluated through safety and efficacy reporting in the UK
Source: Sci Rep. 2022 Nov 7;12:18859. doi: 10.1038/s41598-022-23581-3 (PMC9640588; doi:10.1038/s41598-022-23581-3)
Supplement: Supplementary file 1 — Supplementary Information. [file 41598_2022_23581_MOESM1_ESM.pdf]

Title

Real-world experience of intravenous ferric derisomaltose evaluated through safety and efficacy reporting in the UK

Authors

Rhona C. F. Sinclair; Sean Nadaraja; Nicholas A. Kennedy; Mai Wakatsuki; Sunil Bhandari*

***Corresponding author**

Sunil Bhandari, Department of Renal Medicine, Hull University Teaching Hospitals NHS Trust, Kingston upon Hull, HU3 2JZ, UK

Tel: +44 7714 986 026

Email: sunil.bhandari@nhs.net

Supplemental data

Supplemental figure S1: Age and gender distribution of patients receiving intravenous iron

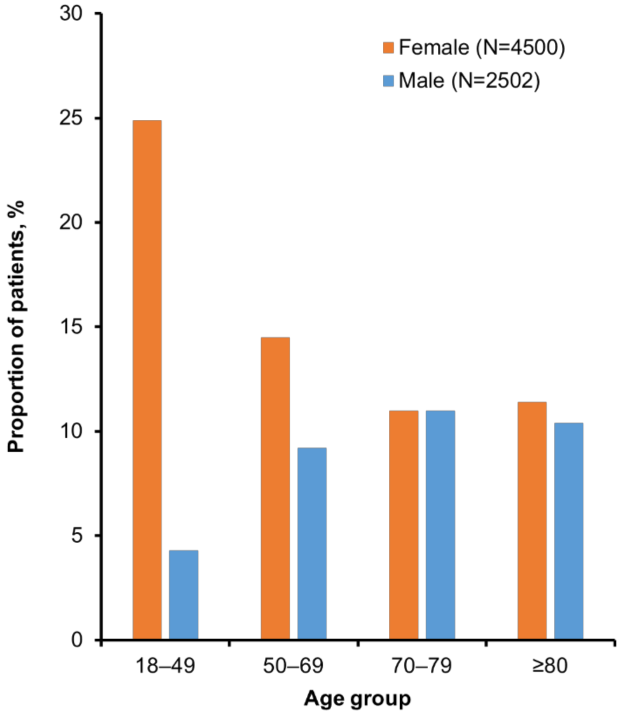

Of the 'All intravenous iron patients' group (N=7354), age data were missing for 236 patients and gender data were missing for 352 patients. The proportions presented in the graph were calculated using the group of patients for whom gender was known (N=7002) as the denominator.

Supplemental figure S2: Proportion of patients experiencing adverse reactions over time from 2014 to 2021

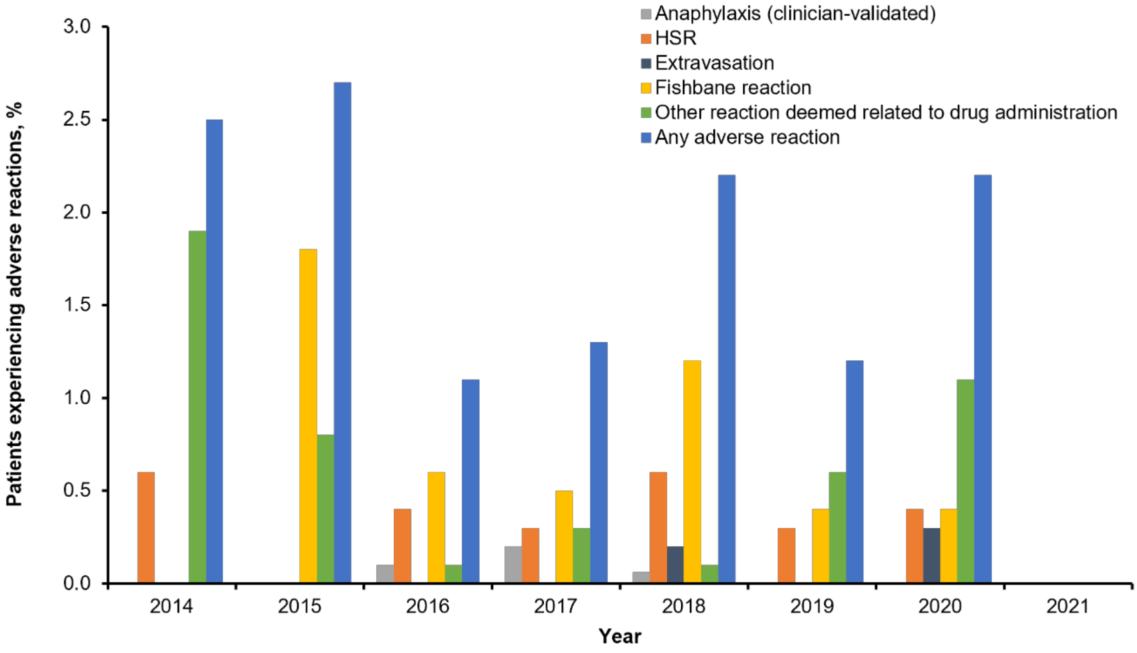

No. of patients receiving intravenous iron	161	603	839	1236	1579	2176	717	54

HSR, hypersensitivity reaction.

Supplemental table S1: Patient disposition, by hospital centre

	All patients screened (N=8058)		
**Hospital centre**	**Patients >18 years, with age data, n (%)**	**Patients not dosed, n (%)**	**Patients in intravenous iron population, n (%)**
Altnagelvin	58 (0.7)	0 (0.0)	58 (0.7)
Antrim (IBD)	117 (1.5)	1 (<0.1)	116 (1.4)
Antrim Area Hospital (OBGYN)	299 (3.7)	2 (<0.1)	297 (3.7)
Gloucester Royal (Renal)	114 (1.4)	0 (0.0)	114 (1.4)
Gloucester Royal (Surgery)	111 (1.4)	2 (<0.1)	109 (1.4)
Hull University	938 (11.6)	0 (0.0)	938 (11.6)
Manchester	120 (1.5)	0 (0.0)	120 (1.5)
Morriston	335 (4.2)	0 (0.0)	335 (4.2)
Princess of Wales	91 (1.1)	0 (0.0)	91 (1.1)
Queen Alexandra	414 (5.1)	0 (0.0)	414 (5.1)
Queen's Romford	2454 (30.5)	0 (0.0)	2454 (30.5)
Royal Devon and Exeter	542 (6.7)	26 (0.3)	516 (6.4)
Royal National Orthopaedic	286 (3.5)	121 (1.5)	165 (2.0)
Royal United Bath	256 (3.2)	8 (<0.1)	248 (3.1)
Royal Victoria Infirmary	155 (1.9)	8 (<0.1)	147 (1.8)
Russell's Hall	50 (0.6)	0 (0.0)	50 (0.6)
University Hospital Southampton	952 (11.8)	45 (0.6)	907 (11.3)
West Middlesex	75 (0.9)	0 (0.0)	75 (0.9)
Wrexham Maelor	200 (2.5)	0 (0.0)	200 (2.5)

'Intravenous iron population' represents all patients who received at least one dose of FDI treatment.

FDI, ferric derisomaltose; IBD, inflammatory bowel disease; OBGYN, obstetrics and gynaecology.

Supplemental table S2: Baseline characteristics, by anaemia status at baseline

	Anaemic (N=6217)	Non-anaemic (N=682)
**Age, years**	(n=6007)	(n=658)
Mean (SD)	60.8 (20.7)	54.8 (18.5)
**Age group, n (%)**	(n=6007)	(n=658)
18–49 years	1974 (31.8)	272 (39.9)
50–69 years	1376 (22.1)	201 (29.5)
70–79 years	1302 (20.9)	121 (17.7)
≥80 years	1355 (21.8)	64 (9.4)
**Weight, kg**	(n=2688)	(n=275)
Mean (SD)	76.9 (19.4)	74.5 (20.3)
**Haemoglobin (g/L)**	(n=6217)	(n=682)
Mean (SD)	96.6 (15.6)	126.9 (9.6)
**Ferritin (µg/L)**	(n=5225)	(n=622)
Mean (SD)	65.1 (155.4)	44.3 (94.4)

The proportions presented in the table were calculated using the overall number of patients as the denominator for each group (N=6217 for anaemic patients; N=682 for non-anaemic patients).

Anaemia at baseline was defined as a haemoglobin level <130 g/L for men, <120 g/L for women, and <105 g/L for obstetrics patients.

N, number of patients; n, number of patients with data; SD, standard deviation.

Supplemental table S3: Total dose of FDI and number of infusions, according to baseline haemoglobin level

Baseline haemoglobin	>130 g/L (N=192)	>120 to ≤130 g/L (N=615)	>110 to ≤120 g/L (N=1144)	>100 to ≤110 g/L (N=1432)	>90 to ≤100 g/L (N=1439)	>80 to ≤90 g/L (N=1116)	≤80 g/L (N=1012)
**Total FDI dose, mg**							
Mean (SD)	1154.7 (430.7)	1287.5 (367.5)	1250.0 (355.7)	1231.0 (325.2)	1260.2 (346.0)	1324.4 (353.5)	1346.6 (340.2)
**Infusions per patient, n (%)**							
1	192 (100)	613 (99.7)	1139 (99.6)	1423 (99.4)	1412 (98.1)	1087 (97.4)	989 (97.7)
2	0 (0.0)	2 (0.3)	5 (0.4)	9 (0.6)	27 (1.9)	29 (2.6)	23 (2.3)

Data are for first course only.

FDI, ferric derisomaltose; N, number of patients; SD, standard deviation

Supplemental table S4: Data parameters

Parameter	Alternative measure to allow for generation of the necessary parameter	Notes
**Demographics**		
Age (years)	Date of birth	Allows for stratification by age during analysis
Gender (male/female)		
Weight (kg)	Dose of FDI (mg) and dosing regimen	
Ethnicity		
**Clinical information**		
Hospital specialty: nephrology, surgery, obstetrics, IBD, other (to be specified)		Can be determined, somewhat, by the data provided and may be grouped accordingly after data collected
Aetiology of anaemia (where known): gastroenterology, IBD, obstetrics, haematology, renal, perioperative, other (to be specified)		
Classification of anaemia: iron deficiency, inflammatory, mixed		
Cancer/non-cancer patients		To allow demonstration of clinical practice of prescribing to patients with cancer, and to demonstrate safety in this subgroup
**Laboratory data**		
Baseline haemoglobin		Essential
Date of baseline haemoglobin (dd/mm/yy)		To calculate mean (range) time between measurements and FDI
Baseline ferritin		
Baseline TSAT		
Baseline CRP		
Baseline creatinine		
Post-treatment haemoglobin		Can be used to perform a time-based analysis if sufficient data are available (not always the case)
Date of post-treatment haemoglobin		

Blood transfusion (yes/no)		Potentially only available, and relevant, for perioperative patients
Date of blood transfusion		
Number of units of blood transfused		
**Safety outcome data**		
Adverse reaction within 24 hours of receiving FDI infusion (yes/no)	If no, move to the next set of questions	
Anaphylaxis (yes/no)		
Anaphylaxis: severity	Mild, moderate, severe	
Anaphylaxis: system affected	Cardiovascular, respiratory, skin	
Fishbane reaction (yes/no)		
Extravasation (yes/no)		
Other reaction		
Dose of FDI administered		To allow ranges for the UK to be determined, and to calculate 'correct/ideal or low dose' based on different regimens
Concurrent blood transfusion administered (yes/no)		
Infusion completed after the reaction (yes/no)		

CRP, C-reactive protein; FDI, ferric derisomaltose; IBD, inflammatory bowel disease; TSAT, transferrin saturation.

Supplemental table S5: Anaphylaxis follow-up questionnaire

**Follow-up questions regarding reported cases of local use of FDI (previously, iron isomaltoside 1000) in your clinical practice**
Your patient data submission included entries with the term ‘anaphylaxis’. We would like to understand this further. Please could you fill out one copy of this form for each of your patients described as having anaphylaxis.
**Interventions**<br>• Was adrenaline administered?<br>• Did the patient require oxygen?<br>• Did the patient complete the infusion?<br>• Did the patient’s symptoms resolve without pharmacological intervention?
**Symptoms**<br>• Did the patient display any of the following isolated symptoms:<br>o Urticaria<br>o Itching<br>o Rash<br>o Mild hypo/hypertension<br>o Tachycardia<br>o Nausea<br>o Headache<br>• Did the patient’s symptoms involve:<br>o Persistent hypotension<br>o Angioedema of the tongue/airway<br>o Hypotension (defined as a drop of 30 mmHg systolic blood pressure from baseline, or systolic blood pressure <90 mmHg)<br>o Chest pain<br>o Generalised urticaria<br>o Non-airway angioedema<br>o Stridor<br>o Bronchospasm<br>o Vomiting<br>o Abdominal pain
When did symptoms first occur?

FDI, ferric derisomaltose.

Supplemental Acknowledgements

Hospital centre	Principal Investigator
Altnagelvin	Fiona Clarke
Antrim (IBD)	Jackie Kearns
Antrim Area Hospital (OBGYN)	Laura Stewart-Maunder
Gloucester Royal (Renal)	Sherry Masoud
Gloucester Royal (Surgery)	Henry Murdoch
Hull University	Sunil Bhandari
Manchester	Rachel Brown
Morriston	Aled Richards
Princess of Wales	Rhys Williams
Queen Alexandra	Alex Pettipher
Queen's Romford	Alison Brownell
Royal Devon and Exeter	Nick Kennedy
Royal National Orthopaedic	Sean Nadaraja
Royal United Bath	Sue Scott
Royal Victoria Infirmary	Rhona Sinclair
Russell's Hall	Adrian Jennings
University Hospital Southampton	Mai Wakatsuki
West Middlesex	Monique Chituku
Wrexham Maelor	Sharon Owen

IBD, inflammatory bowel disease; OBGYN, obstetrics and gynaecology.
